# Response: Potential dose variability for small‐field plans delivered with Elekta Agility collimators

**DOI:** 10.1002/acm2.13414

**Published:** 2021-09-04

**Authors:** Friedlieb H. Lorenz, Matthew I. Paris

**Affiliations:** ^1^ Department of Radiation Oncology Southern District Health Board Dunedin New Zealand

Dear Editor,

We read with interest the letter to the editor written by Foy et al.^1^ regarding a recently published article on 6 FFF beams delivered on an Agility multileaf collimator (MLC).^2^

We appreciate the interest in this publication by the authors; however, after careful review we have identified several apparent misunderstandings in the letter to the editor by Foy et al. The assertions presented by Foy et al. do not specifically pertain to our article as the data discussed in their letter do not relate directly to the purpose of the cited article.^2^

Specifically, we would like to correct the following misinterpretations by Foy et al.:

1. The plans for the patient‐specific quality assurance (PSQA) performed by Foy et al. for their own flattening filter free (FFF) and flattened (FF) beams were calculated in Pinnacle TPS. This differs from the treatment planning system (TPS) used in Lorenz and Paris^2^ (Elekta's Monaco TPS). If the Agility leaf constraint curve is implemented correctly in Pinnacle, the findings described in Lorenz and Paris might not be of great relevance for Pinnacle users.
2. Foy et al. must have misinterpreted some findings in Lorenz and Paris^2^ as they stated in their letter “The authors concluded that the reduced passing rate for plans using FFF was due to the operation of the Agility MLC and its ability to violate leaf position tolerances.”^1^ Such a conclusion had never been reached in Lorenz and Paris,^2^ where it was clearly stated that there are other factors involved in failing QA measurements: “The linear regression shown in Figure 5 suggests that there could be some correlation between passing rates and ratio of small segments in any given plan. The relatively low *R* ^2^ values of 0.2229 and 0.436 seem to indicate that there are most likely other factors involved causing such poor passing rates, which warrant further, more systematic investigations.”^2^
3. We agree with Foy et al. that “Agreement in calculated and measured doses can vary with fluctuations in daily output, linac limitations, complexity of the measured plan, and a myriad of many other factors that have been extensively investigated.”^1^However, Foy et al. imply that Lorenz and Paris’ conclusion might have been that “differences in calculated and measured doses and the effect on PSQA passing rates are solely due to the operation of the Agility™ MLC.”^1^ As described under (2), this conclusion had not been reached. This was made clear in the title of said article as well as in the discussion: “Depending on the location of these 3.5 mm gaps, they could introduce a relatively large error in delivered dose.”^2^
4. We also agree with Foy et al.’s statement regarding the effects of small MLC gap sizes: “If plans are created with gap sizes smaller than 5 mm, then the calculated dose distributions will differ from those that can be delivered with an Agility™ MLC based on these limitations.”^1^ It was shown by Lorenz and Paris that Monaco's sequencer can generate a relatively high proportion of 3.5 mm leaf apertures in complex VMAT plans.^2^
  The extent to which small gaps exist and affect a facility's assemblage of plans will depend upon the TPS and plan complexity. The assumption by Foy et al. that “3.5 mm will not necessarily be found in most clinical plans”^1^may not be universally valid.
5. Foy et al. have compared their PSQA results for FFF and FF beams and concluded that significant differences exist between those two modalities. They have stated that this is most likely related to beam model and commissioning issues. While this could be so for their own beam model in Pinnacle, no inference can be made to the results published in Lorenz and Paris.
  More importantly, Foy et al. seem to confuse PSQA results with the measurement results of the specifically designed MLC fields, as they are suggesting that the “reported issues are not within the MLC design.”^1^ It was shown in Figures 2 and 3 in Lorenz and Paris^2^ that the leaf positions are modified during delivery for the Agility MLC, which could indeed be an issue related to the MLC design.
6. Foy et al. are suggesting that “unnecessarily alarming language”^1^ has been used in Lorenz and Paris,^2^ without providing evidence to justify this assertion. We therefore consider this to be an unsubstantiated criticism.
  As already stated in Lorenz and Paris, more work is needed to fully characterize the performance of the Agility MLC including leaf positioning tolerances in dynamic delivery mode, and the impact of potential positioning inaccuracies on PSQA.
